# Poor outcome of comprehensive therapy in a case of laryngeal synovial sarcoma

**DOI:** 10.2478/raon-2013-0027

**Published:** 2013-05-21

**Authors:** Yang-Yang Bao, Quin-Ying Wang, Shui-Hong Zhou, Kui Zhao, Ling-Xiang Ruan, Hong-Tian Yao

**Affiliations:** 1 Department of Otolaryngology, Zejiang University, Hangzhou, Zhejiang, China; 2 Department of PET/CT centre, Zejiang University, Hangzhou, Zhejiang, China; 3 Department of Radiology, Zejiang University, Hangzhou, Zhejiang, China; 4 Department of Pathology, The First Affiliated Hospital, College of Medicine, Zejiang University, Hangzhou, Zhejiang, China

**Keywords:** synovial sarcoma, larynx, PET/CT, follow up

## Abstract

**Background:**

Synovial sarcoma is common in the extremities. Our search revealed only 17 cases of synovial sarcoma of the larynx in the English-language literature.

**Case report:**

We report an additional case of a 37-year-old man with primary laryngeal synovial sarcoma who underwent positron emission tomography/computed tomography (PET/CT) following the treatment. Although the patient received comprehensive therapy including surgery, radiotherapy, repeated chemotherapies, and targeted therapies, he had an unfavourable outcome and died of distant metastases.

**Conclusions:**

In synovial sarcoma of the larynx, PET/CT can detect recurrence and metastasis. PET/CT can also predict the treatment effect in patients with synovial sarcoma.

## Introduction

Synovial sarcoma is common in the extremities.^1^ Only 3–9% of all cases of synovial sarcoma occur in the head and neck region, and the least frequent site of occurrence is the larynx.^2^,^3^ Our search revealed only 17 cases of synovial sarcoma of the larynx in the English-language literature.^2^–^18^

Since few cases of laryngeal synovial sarcoma have been reported, its histogenesis, natural history, optimal treatment strategy, and long-term prognosis are unknown. Most primary synovial sarcomas of the head and neck metastasise to the lungs; among these, only a few cases were reported to have cervical metastases.^19^ The local recurrence rate of synovial sarcoma is 8–60%. Tumours usually recur within 2 years of the initial therapy.^20^ The diagnostic workup for recurrence or metastasis of synovial sarcoma involves conventional imaging, including computed tomography (CT) and magnetic resonance imaging (MRI).^21^ During the past decade, 18F-fluorodeoxyglucose (18FDG) positron emission tomography (PET)/CT has become an adjunct tool to conventional imaging in the staging and follow-up of sarcoma.^22^ A few reports have described the use of PET/CT for synovial sarcoma^1^,^20^,^23^, and no report has presented the PET/CT features of laryngeal synovial sarcoma.

In the majority of cases, the surgical excision with a wide margin is the first treatment choice. The effect of chemotherapy or radiotherapy is controversial.^24^–^26^ We report a case of a patient with primary laryngeal synovial sarcoma who underwent PET/CT following treatment. Although the patient received comprehensive therapy including surgery, radiotherapy, repeated chemotherapies, and targeted therapies, he had an unfavourable outcome and died of whole-body metastasis in November 2011.

## Case report

**FIGURE 1:**
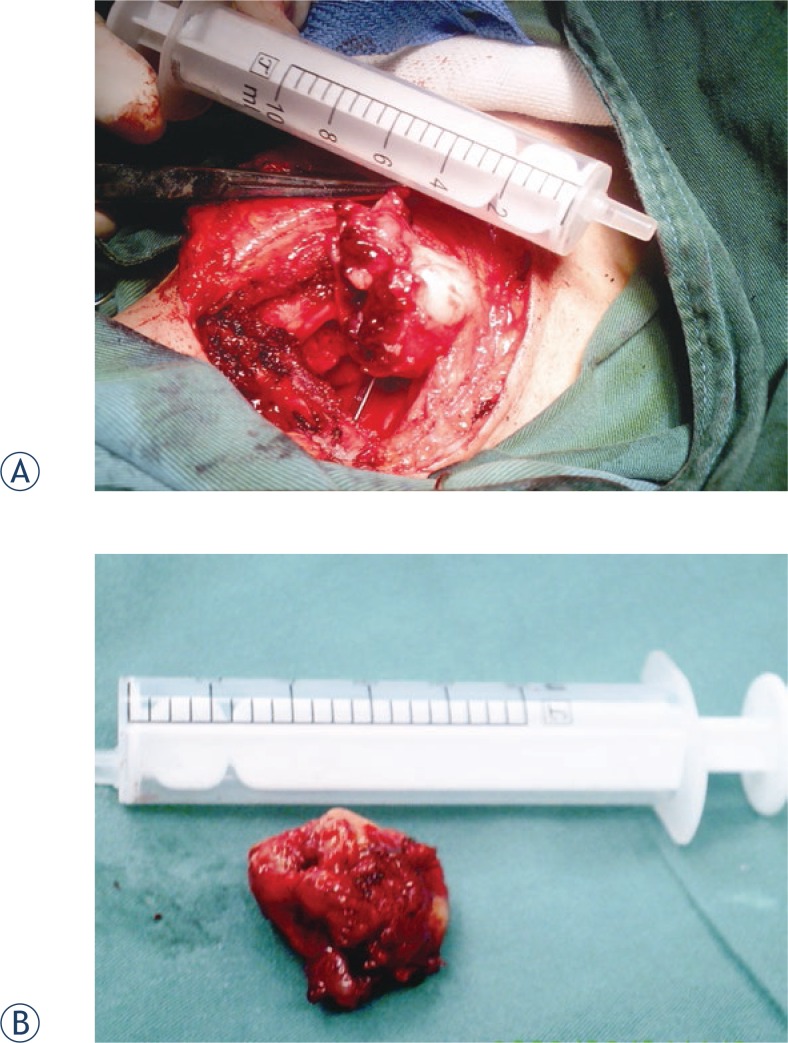
(a) The mass was tan-red and irregular and had an ulcer covered by blood clots and a fleshy cut surface. (b) Surgical sample.

On 4 June 2008, a 37-year-old man was referred to our department due to a 1-month history of sore throat and blood in the phlegm. He also complained of dysphagia. He denied hoarseness and respiratory dyspnoea. His medical history was unremarkable. Laryngoscopy showed a 2 × 2.5 cm wine-coloured tumour in the right aryepiglottic fold that involved the inner wall of the right piriform sinus. The surface was covered with blood clots. The motion of the bilateral vocal cords and the remainder of the larynx appeared normal. Cervical lymphadenopathy was absent. CT revealed a 3.5 × 2.2 cm irregular soft-tissue mass in the right aryepiglottic fold extending to the right piriform sinus, with low to moderate heterogeneous enhancement after the injection of contrast medium. No enlarged node was present. Under general anaesthesia (a tracheotomy was performed), biopsy was performed by suspension laryngoscopy. The frozen section results suggested that the tumour was a spindle cell tumour. The tumour was removed completely *via* lateral cervical incision and partial laryngectomy. The laryngeal function was preserved. During the operation, we found that the tumour was located in the right supraglottic area, with a pedicle in the right aryepiglottic fold extending to the right piriform sinus. Mass was tan-red and irregular and had an ulcer covered by blood clots and a fleshy cut surface (Figure 1).

**FIGURE 2:**
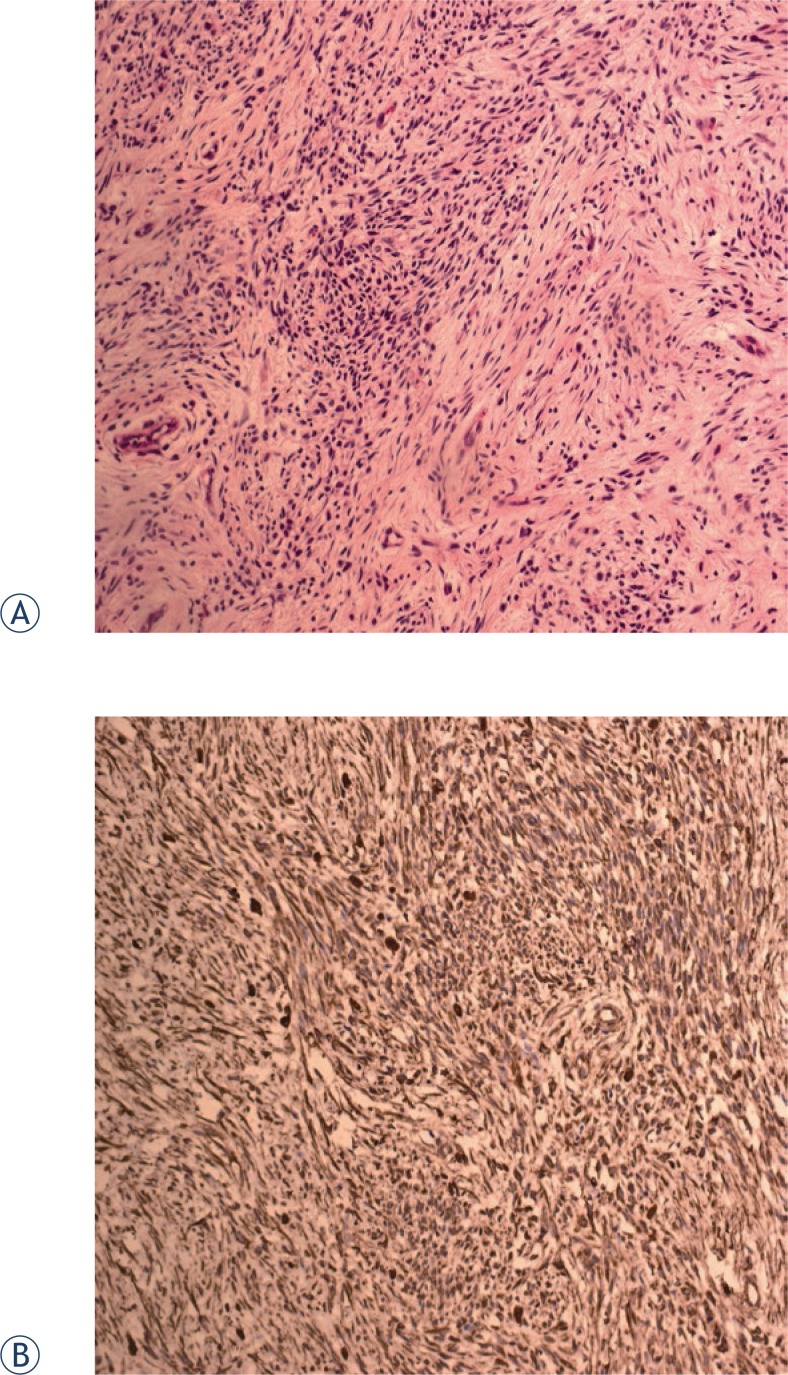
(a) Pathology showed that the tumour consisted of small, uniform spindle cells invading the surrounding muscles (HE×20). (b) Immunohistochemical examination for vimentin was positive (EliVision×20).

The postoperative pathological results showed that the tumour consisted of small, uniform spindle cells invading the surrounding muscles. The immunohistochemical examination for vimentin and CD99 was positive. Epithelial membrane antigen was focal positive. These results led to a diagnosis of monophasic synovial sarcoma. The resection margins were tumour free (Figure 2).

**FIGURE 3:**
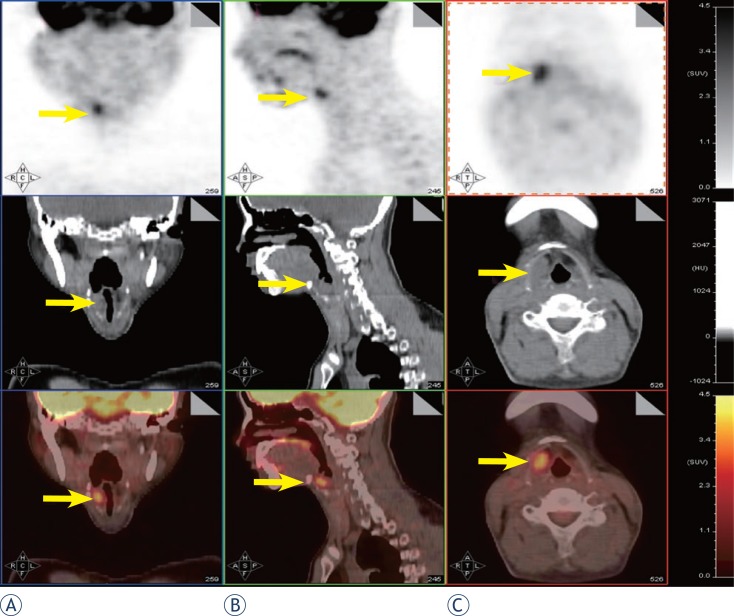
PET/CT showed high FDG uptake in the region of the right hypopharynx and laryngeal inlet (SUV_max_ = 4.1) and no distant metastasis.

Postoperative radiotherapy was given; the total dose was 66 Gy. On 26 October 2009, MRI of the head and neck revealed a 1 × 2 cm mass in the region of the right hypopharynx and laryngeal inlet. On 4 November 2009, 18FDG-PET/CT demonstrated high FDG uptake coincident with MRI findings (standardised uptake value [SUV]_max_ = 4.1) and no distant metastases (Figure 3). A partial laryngopharyngectomy was performed. After surgery, the patient was followed regularly at 2-month intervals with CT or MRI examination of the head and neck.

**FIGURE 4:**
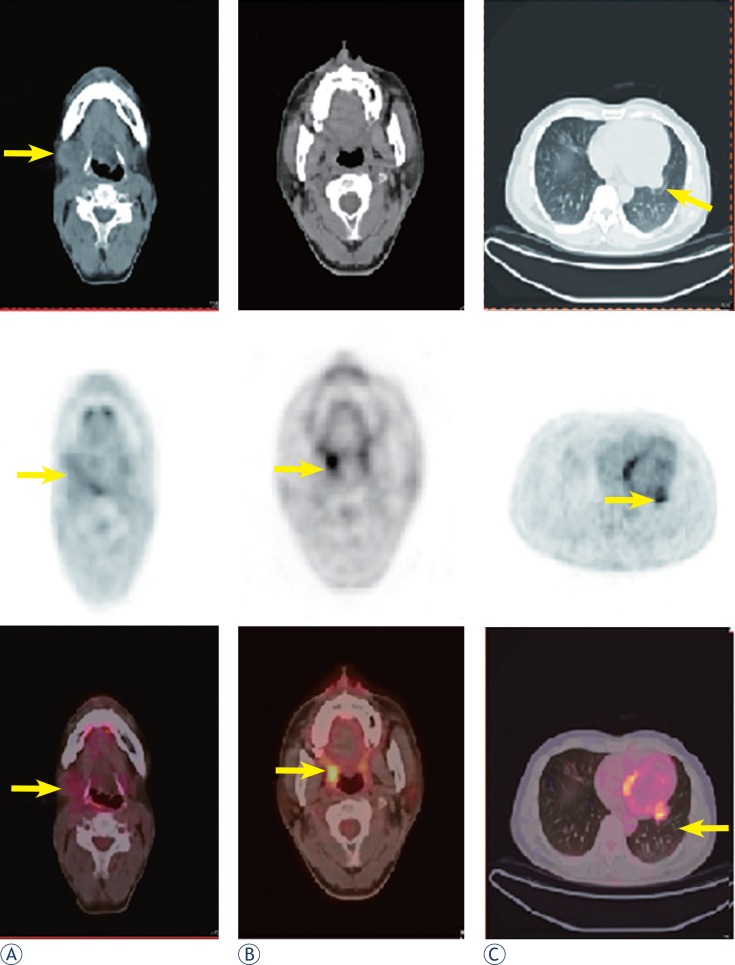
(a, b) Nine months after the second surgery, PET/CT revealed high FDG uptake in the right submaxillary lymph node (SUV_max_= 3.2), right oropharynx (SUV_max_= 5.4) and(c) multiple nodules in both lungs (SUV_max_= 4.6).

**FIGURE 5:**
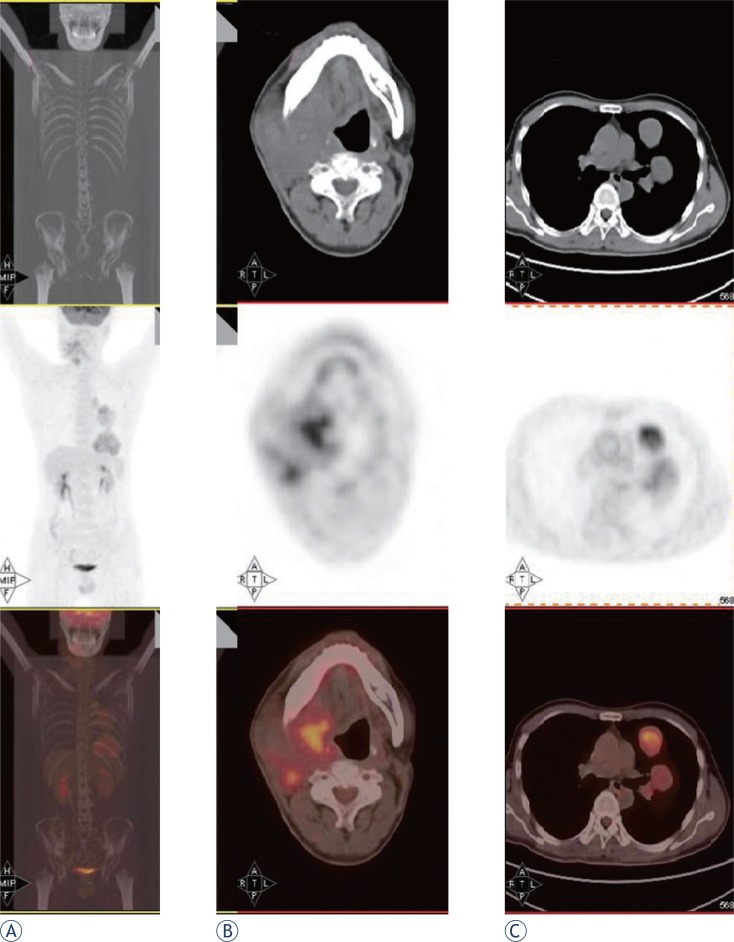
After radiotherapy, repeated chemotherapies and targeted therapies, (a) a whole body scan showed FDG uptake in the right neck and lung. (b) PET/CT revealed a 7.5 × 9-cm mass in the right submaxillary region with high FDG uptake (SUV_max_ = 5.2) involving the right parotid gland, right tongue base, right mandible, jugular vein, carotid artery, and surrounding muscles. (c) Multiple nodules in both lungs, were evident with the largest being up to 6.68 cm in diameter (SUV_max_= 6.02).

In August 2010, CT of the head and neck revealed a 3.6 × 2.6 cm mass in the right submaxillary region. Further CT of the lungs revealed bilateral lung metastases. PET/CT revealed high FDG uptake in the right submaxillary lymph node (SUV_max_ = 3.2), right oropharynx (SUV_max_ = 5.4), and multiple nodules in the bilateral lungs (SUV_max_ = 4.6; Figure 4). These results suggested local recurrence and cervical lymph node and lung metastases. The patient received chemotherapy and concurrent targeted treatment comprising adriamycin (40 mg/m^2^, days 1–2), ifosfamide (2 g/m^2^, days 1–4), dacarbazine (300 mg/m^2^, days 1–4), and anti-epidermal growth factor receptor monoclonal antibody (nimotuzumab, 200 mg/m^2^, days 1–4). The patient underwent three cycles of chemotherapy at 3-week intervals. On 18 November 2010, the first-line therapy package was completed. The cervical mass was treated with intratumoural injection of recombinant adenovirus p53 agent injection once a week for 4 weeks and another four cycles of nimotuzumab (200 mg) at 1-week intervals. CT demonstrated no regression of the metastatic cervical lymph node and lung lesions. In February 2011, the patient underwent additional chemotherapy comprising docetaxel (110 mg, day 1), cisplatin (40 mg, days 1–3), and rh-endostatin (Endostar) (15 mg, days 1–14) every 21 days for up to five cycles. The lesions were not controlled. In July 2011, the chemotherapy was changed to cetuximab (600 mg, day 1) every week for up to three cycles. CT of the lungs and MRI of the head and neck showed that the lesions were larger. On 22 September 2011, PET/CT revealed a 7.5 × 9 cm mass in the right submaxillary region with high FDG uptake (SUV_max_ = 5.2) involving the right parotid gland, right tongue base, right mandible, jugular vein, carotid artery, and surrounding muscles, and multiple nodules in the bilateral lungs (largest = 6.68 cm in diameter; SUV_max_ = 6.02; Figure 5). The cervical mass was treated by local cryoablation, but did not regress. In 11 November 2011, the patient died of brain metastases.

## Discussion

Only 3–9% of all cases of synovial sarcoma occur in the head and neck. The least frequent site of occurrence is the larynx. To our knowledge, only 18 cases have been reported (including the current case) (Table 1).^2^–^18^ The reported cases included 13 males, three females, and one case in which the patient’s sex was not reported.^2^–^18^ The male to female ratio was about 4:1. The age of the patients ranged from 12 to 79 years at initial presentation, with a mean age of 37 years (data were not available for four patients).^2^–^18^ Of the 17 patients for whom data were available^2^–^7^,^9^–^18^, 16 (94.1%) had tumour located in the supraglottic and 1 (5.9%) had tumour in the subglottis; no patient had tumour in the glottis area. For the 16 patients for whom follow-up data were available, the follow-up times ranged from 3 months to 16 years.^2^–^6^,^8^–^11^,^13^–^18^ Of these 16 patients, only four (including our patient) developed recurrence and three developed distant metastases. Only two patients (including our patient) died of the disease. The poor outcome might be related to the large extent of the laryngeal synovial sarcoma.^18^

In the majority of cases, the surgical excision with a wide margin is the first treatment choice for synovial carcinoma. The effect of chemotherapy or radiotherapy is controversial.^25^–^27^ The main treatment regimen for laryngeal synovial sarcoma is surgery, including laryngopharyngectomy, hemilaryngectomy, tumourectomy, total laryngectomy, or tumour resection using a CO_2_ laser (for localised lesions).^3^–^18^ Among the patients who underwent CO_2_ laser surgery, no recurrence or metastasis occurred, and the disease-free survival times were 2 years^11^, 3 years^8^, and 15 months.^5^ The favourable results in this group might be associated with the low volume of the tumours subjected to CO_2_ laser surgery.^5^,^8^,^11^ In cases of laryngeal synovial sarcoma (excluding our case and one case reported by Gatti *et al*.^18^), postoperative chemo/radiotherapy seemed to be effective.^2^,^4^,^11^,^14^–^16^ No recurrences or metastases occurred, and the longest survival time was 16 years.^15^ Chemo/radiotherapy also seemed to be useful in the treatment of distant metastases and local recurrence.^10^,^14^ Some reports had a very short follow-up period (<2 years), and the exact outcomes require a further investigation.^2^,^4^–^6^,^9^,^11^,^13^ Although our case involved multiple therapeutic strategies, including surgery, radiotherapy, repeated chemotherapies, and targeted therapies, the outcome was poor. This result was similar to that reported by Gatti *et al*.^18^ Consequently, the ideal treatment has yet to be established because of the limited number of available reports on laryngeal synovial sarcoma.

Magnetic resonance imaging is often recommended as a follow-up modality.^21^,^27^ Recent studies have indicated that PET is useful in the follow-up of synovial sarcoma.^1^,^23^ PET/CT has also been used as a diagnostic tool for synovial sarcoma, as well as lymphoma.^20^,^28^,^29^ Charest *et al*. demonstrated 80.0% sensitivity of PET/CT in 20 soft-tissue synovial sarcomas with a mean SUV_max_ of 10.9.^28^ Erturhan *et al*. reported the use of PET/CT for diagnosis and follow-up in a case of kidney synovial sarcoma. They found slight FDG uptake in the synovial sarcoma and in multiple lymph nodes (SUV_max_ = 3.5). PET/CT detected no differentiation in multiple lymph nodes in the fourth postoperative month; however, CT did not show these lymph nodes, and they were reported as normal.^23^ Lisle *et al*. assessed 44 patients with synovial sarcoma before therapy and resection by FDG-PET. They found that the pre-treatment tumour SUV_max_ predicted the overall and progression-free survival. Patients with SUV_max_ >4.35 had reduced disease-free survival and were therefore at high risk for local recurrence and metastatic disease.^1^ In our case, although the patient did not undergo pre-treatment PET/CT, three repeated post-treatment PET/CT examinations demonstrated the unsatisfactory treatment outcomes. In this case, SUV_max_ did not decrease in response to the various treatments. These findings were consistent with the results of Lisle *et al*.^1^ Therefore, PET/CT can be a useful workup tool for synovial sarcoma.

## Conclusions

Here, we report a case of laryngeal synovial sarcoma. Although the patient received a comprehensive therapy including surgery, radiotherapy, repeated chemotherapies, and targeted therapies, he had an unfavourable outcome and died of whole-body metastases. Since cases involving the larynx are extremely rare, the treatment of laryngeal synovial sarcoma should follow the guidelines for other tumour sites. To our knowledge, this is the first report of PET/CT findings of laryngeal synovial sarcoma. PET/CT can detect local recurrence and metastasis of laryngeal synovial carcinoma. PET/ CT can also predict the treatment effect in patients with synovial sarcoma.

## Figures and Tables

**TABLE 1:** English-Literature review of synovial sarcomas of larynx

Pt.No/Ref	Sex/Age	location	treatment	Follow-up
2 Al-Nemer A (2011)^2^	M/26	Larynx	Surgery+radiotherapy	20m, NED
3 Fernández-Aceñero (2009)^4^	M/12	Supraglottic	Chemotherapy+ Local resection	4m NED
4 Capelli (2007)^5^	M/57	Supraglottic	CO 2 laser cordectomy	14m NED
5 Mhawech-Fauceglia (2007)^6^	M/79	Supraglottic	a wide-field total laryngectomy	3m NED
6 Abou Zeid (2006)^7^	M/26	Supraglottic	excision of the supraglottic tumor with CO(2) laser surgery	NA
7 Boniver (2005)^8^	NA	Right aryepiglottic fold	CO 2 laser resection	36m NED
8 Szuhai (2004)^9^	M/54	Supraglottic	laryngopharyngectomy a left-sided modified neck dissection	24 m NED
9 Bilgic (2003)^10^	M/24	Supraglottic	hemilaryngectomy	12m, local recurrence, total laryngectomy+radiotherapy 20m, lung metastasis, chemotherapy, 42m NED
10 Papaspyrou (2003)^11^	M/16	Supraglottic	CO 2 laser+radiotherapy	24m NED
11 Taylor (2002)^12^	F/68	Cricoids cartilage	total laryngopharyngectomy, cervical esophagectomy, bilateral neck dissection,	NA
12 Dei Tos (1998)^13^	M/27	Supraglottic	Surgery	3m local recurrence, chemotherapy+radiotherapy, 3m, hemilarygectomy, 9m NED
13 Morland (1994)^14^	M/14	Left arytenoid	Tumorectomy, Recurrence after 3 years Total laryngectomy, CT, radiotherapy	10m NED
14 Ferlito (1991)^15^	M/28	Supraglottic	Supraglottic laryngectomy+right neck dissection+radiotherapy	16y NED
15 Pruszczynski (1989)^16^	F/28	Right aryepiglottic fold and false cord	Tumorectomy, radiotherapy	3y NED
16 Quinn (1984)^17^	M/76	Right subglottic area	Frontolateral laryngectomy	3y NED
17 Gatti (1975)^18^	F/28	Left hemilarynx hypopharynx	Pharyngolaryngectomy,	1 year, lung metastasis, radiotherapy+chemotherapy, 2.5 year died
18 Miller (1975)^3^	F/23	Interarytenoid and left arytenoids area	Supraglottic laryngectomy total laryngopharyngectomy	12y NED
**Present case**	**M/37**	**Supraglottic**	**Surgery+chemo.-radiotherpay**	**28m local recurrence; 41m, Died of metastasis**

Ref = number of reference; NED = no evidence of disease; NA = not available
